# Adverse intraoperative events during surgical repair of ruptured cerebral aneurysms: a systematic review

**DOI:** 10.1007/s10143-020-01312-4

**Published:** 2020-06-16

**Authors:** William R. Muirhead, Patrick J. Grover, Ahmed K. Toma, Danail Stoyanov, Hani J. Marcus, Mary Murphy

**Affiliations:** 1 Department of Neurosurgery, The National Hospital for Neurology and Neurosurgery, Queen Square, London, WC1N 3BG UK; 2 Wellcome/EPSRC Centre for Interventional and Surgical Sciences, University College London, London, UK

**Keywords:** Aneurysm clipping, Intraoperative complications, Adverse events, Subarachnoid haemorrhage

## Abstract

Compared with endovascular techniques, clipping of ruptured cerebral aneurysms has been shown to associate with increased morbidity in several studies. Despite this, clipping remains the preferred option for many aneurysms. The objective of this study is to describe the reported adverse events of open repair of ruptured cerebral aneurysms and their impact on patient outcome. The PubMed, Embase and Cochrane databases were searched between June 1999 and June 2019 to identify original studies of at least 100 patients undergoing surgical repair of ruptured cerebral aneurysms and in which adverse event rates were reported. Thirty-six studies reporting adverse events in a total of 12,410 operations for repair of ruptured cerebral aneurysms were included. Surgical adverse events were common with 36 event types reported including intraoperative rupture (median rate of 16.6%), arterial injury (median rate of 3.8%) and brain swelling (median rate 5.6%). Only 6 surgical events were statistically shown to associate with poor outcomes by any author and for intraoperative rupture (the most frequently analysed), there was an even split between authors finding a statistical association with poor outcome and those finding no association. Even with modern surgical techniques, the technical demands of surgical aneurysm repair continue to lead to a high rate of intraoperative adverse events. Despite this, it is not known which of these intraoperative events are the most important contributors to the poor outcomes often seen in these patients. More research directed towards identifying the events that most drive operative morbidity has the potential to improve outcomes for these patients.

**Electronic supplementary material:**

The online version of this article (10.1007/s10143-020-01312-4) contains supplementary material, which is available to authorized users.

## Introduction

Patients with ruptured cerebral aneurysms which are suitable for endovascular repair have been shown to have a reduced risk of death and dependency at 1 year when treated endovascularly compared with open surgical techniques [1, 2]. As open surgical repair is associated with a higher rate of aneurysm occlusion in the short and long term, the poor outcomes in the surgical group are believed to be due to the increased morbidity of surgery rather than rebleeding [3]. As many as 43% of patients undergoing surgery for ruptured cerebral aneurysms will experience an immediate postoperative neurological deterioration [4] and in one series of subarachnoid haemorrhage treated with surgical clipping, 36% of the deaths and permanent disabilities were attributed to technical intraoperative complications [5]. The profile of adverse events is very different for surgery for unruptured cerebral aneurysms which have much lower morbidity due to more favourable operating conditions [6]. The aim of this review is to better describe the adverse intraoperative events that occur, specifically in surgery for ruptured cerebral aneurysms which appear to cause significant morbidity for this group.

## Materials and Methods

The Preferred Reporting Items for Systematic Reviews and Meta-Analyses (PRISMA) statement was used in the preparation of this manuscript [7] and it was registered with the National Institute of Health Research PROSPERO.

### Search methods

The PubMed, Embase and Cochrane databases were searched over a 20-year period between June 1999 and June 2019. The Boolean search term was used (“intracranial aneurysm” OR “intra-cranial aneurysm” OR “cerebral aneurysm” OR “subarachnoid hemorrhage” OR “subarachnoid haemorrhage” OR “SAH”) AND (“surgery” OR “surgical” OR “neurosurgical” OR “neurosurgery” OR “clipping”) AND (“intraoperative” OR “intra-operative”) AND (“complications” OR “adverse events”). References were reviewed to identify further articles for inclusion. Two authors (WM and PG) independently identified articles using the above search criteria.

### Inclusion and exclusion criteria

Titles and abstracts were screened to identify publications that met the following inclusion criteria: [1] original studies, [2] reporting a series of at least 100 ruptured aneurysms treated with open surgical repair and [3] describing intraoperative adverse events in these patients.

Papers were excluded if they were: [1] published outside the range June 1999 and June 2019, [2] not in English language and [3] only reported intraoperative adverse events with reference to a larger series of patients (e.g. those undergoing endovascular and open repair).

### Data extraction

The following data were extracted from eligible full articles: [1] year of publication, [2] sample size, [3] whether surgery was performed by a self-reported generalist or self-reported cerebrovascular specialist neurosurgeon, [4] special surgical techniques or adjuncts reported, [5] intraoperative complications reported (the definition of “adverse event” is expanded below), [6] frequency of each complication and [8] impact of complications on patient outcome (if reported).

We follow the CLASSIC authors in their work on surgical complications in taking a broad definition of an “adverse event” as “any deviation from the ideal intraoperative course” [9]. Consequently, the categories of complications recorded include: *injuries* such as damage to arteries or other structures; *incompletion* of the surgical goal (in this case invariably failure to secure the aneurysm); *increased operative difficulty* (due to, for example, brain swelling); *interventions* that were additional to the ideal intraoperative course (where there is ambiguity as to whether an intervention was considered part of the ideal course by the operating surgeon—e.g. a short period of temporary clip application—we favoured recording the intervention); *physiological derangements* (e.g. unintentional hypotension or electrolyte imbalance); and *neurological harm* such as intraoperative strokes or deterioration in conscious level. We formally considered the intraoperative period to begin at induction of anaesthesia and end 2 h after the patient left the operating theatre; however, frequently adverse events (such as a neurological deficit or a radiological stroke) were recognised some time after this and in such cases, we followed the publication authors in attributing them to the intraoperative period if that was their assessment.

Where authors reported a graded operative difficulty (e.g. slight, moderate, severe brain swelling or easy, moderate or difficult exposure), we recorded this binarily, with “severe” or “difficult” conditions recorded as complications, and considered the “easy”, “slight” and “moderate” categories to be within normal limits.

### Appraisal of evidence

The Jadad and Methodological Index for Non-randomised Studies (MINORS) scoring systems were used as frameworks to inform evaluation of the quality of randomised and non-randomised studies respectively [10, 11].

## Results

**Fig. 1:**
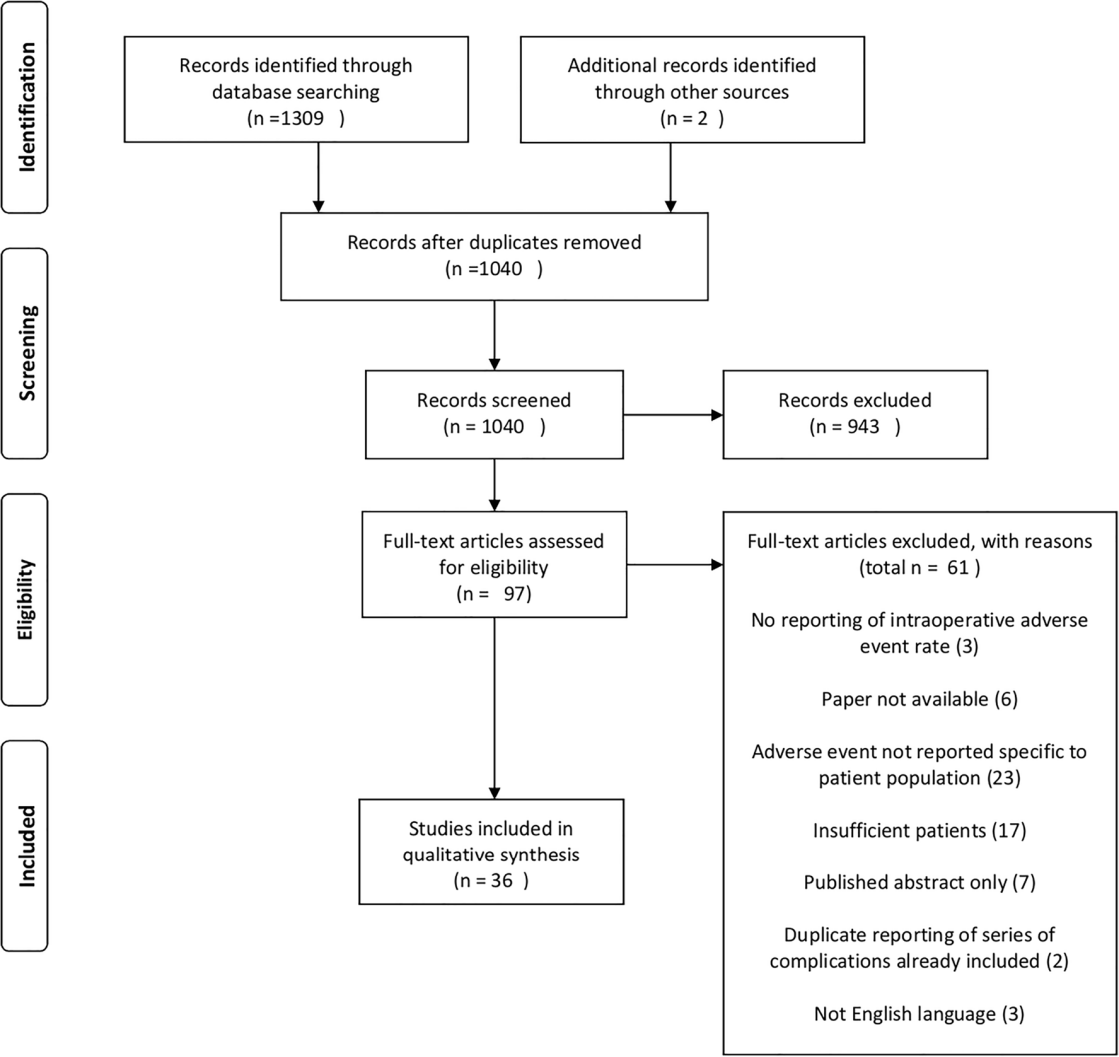
PRISMA flow diagram

After automated elimination of duplicates by the systematic review software (Covidence, Melbourne, Australia), our search identified 1309 references for review. After automated screening for duplicates, this was reduced to 1038 references for review. Two further articles were identified from the authors’ existing knowledge of the literature for a total of 1040 references for screening (Fig. 1). A total of 943 records were excluded because they did not present original data; reported fewer than 100 open surgical repairs of ruptured intracranial aneurysms; did not report intraoperative complications; were limited to published abstract; or were duplicate references not already screened out by the review software. Ninety-seven articles were identified for full-text review. Full-text screening of these articles led to exclusion of a further 61 references. In all, 36 articles were identified that satisfied the inclusion criteria (Table 1). These 36 articles reported adverse events in 12,410 operations [4, 5, 12–21, 23–25, 27, 28, 30–35, 37–49]. Two of these papers [4, 36] both reported the same patient series from the Intraoperative Hypothermia for Aneurysm Surgery Trial—as their focus was on different complication profiles, in these patients, both were included.

**Table 1:** Summary of publication included in this review

Author	Year	Focus of paper	Design	Number of operations for ruptured aneurysms	Specialist or generalist neurosurgeon	Techniques or adjuncts	Impact of complications on outcomes
Ayling [12]	2015	Review of surgical complications from the CONSCIOUS-1 Study into Clazoneten to prevent surgical ischaemia	Post hoc analysis of RCT groups	181	Not reported	None	Induced hypotension and intraoperative hypotension were associated with poor postoperative GCS
Burkhardt [13]	2016	Analysis of preoperative predictors of intraoperative rupture	Retrospective cohort	100	Not reported	Temporary clipping used variably—no rate reported	IOR does not have a significant impact on clinical outcome
Chalouhi [14]	2012	Use of intraoperative angiography to predict surgical revision	Retrospective cohort	360	Not reported	Intraoperative angiography	Ruptured aneurysms were significantly more likely to require adjustment following on-table DSA
Darkwah Oppong [15]	2018	Factors predicting intraoperative aneurysm rupture	Retrospective cohort	365	Not reported	None	IOAR independently predicted unfavourable outcome at 6 months and in-hospital mortality for patients with RIA
Dashti [16]	2009	Assessment of intraoperative ICG	Prospective cohort	112	Not reported	On-table angiography and Doppler	No statistical inference drawn
Dhandapani [17]	2012	Assessment of elective temporary clipping on neurological outcomes	Prospective cohort	273	Not reported	Mannitol given to all patients, elective temporary clipping	No operative rupture, short elective temporary clipping, rescue temporary clip (versus elective) was felt to be protective, total temp clip time > 20 min predicts poor outcome
Doerfler [18]	2018	Incidence and impact of secondary cerebral insults on outcome in subarachnoid haemorrhage	Retrospective cohort	421	Not reported	None	Intraoperative rupture was associated with unfavourable outcome
Elijovich [19]	2008	Predictors of intraprocedural rupture in patients treated for ruptured intracranial aneurysms	Prospective cohort	711	Not reported	None	Intraoperative rupture higher risk of poor outcome (31% worse)
Ferch [20]	2002	Analysis of risk factors for stroke in surgery for subarachnoid haemorrhage	Retrospective cohort	850	Specialist	None	Increased incidence of stroke with prolonged (> 10 min) temporary clipping
Foroohar [21]	2000	Intraoperative variables and outcome after aneurysm surgery	Retrospective cohort	190	Not reported	None	Lower maximum intraoperative systolic blood pressure was associated with good outcome
Fridriksson [5]	2002	Prospective collection of aneurysm complications	Prospective cohort	355	Mixed	Temporary clipping for with neuroprotection for some cases	Intraoperative technical complications caused 8% of deaths and 28% of permanent disabilities
Goertz [22]	2018	Impact of aneurysm shape on rupture during clipping	Retrospective cohort	138	Not reported	None	Intraoperative rupture had no impact on rate of unfavourable outcome
Gu [23]	2018	Using cardiac-gated CT angiography to predictive intraoperative rupture	Prospective cohort	153	Specialist	Cardiac-gated CT used to predict intraoperative rupture	None
Hoff [24]	2008	Impact of intraoperative hypotension on outcomes	Retrospective cohort	164	Not reported	None	Intraoperative hypotension was not demonstrated to be associated with poor outcome
Juvela [25]	2009	Whether apolipoprotein E genotype predicts outcome after aneurysmal subarachnoid haemorrhage	Prospective cohort	102	Not reported	Mannitol in all operations, thiopental and increased BP prior to temporary clipping	Duration of temporary clipping was associated with stroke
Kapsalaki [26]	2008	The role of intraoperative micro-Doppler ultrasound in verifying proper clip placement in intracranial aneurysm surgery	Retrospective cohort	121	Not reported	None	No comment on outcome
Kashkoush [27]	2017	Utility of SSEP in predicting stroke	Retrospective cohort	133	Not reported	SSEP monitoring	SSEP is predictive of stroke
Kivisaari [28]	2004	Investigation of utility of control angiography	Prospective cohort	493	Experienced	None	No correlation with neurological outcome reported
Lakicevic [29]	2015	Impact of intraoperative rupture on outcome	Retrospective cohort	536	Not reported	None	Intraoperative rupture increases rate of postoperative deficit
Leipzig [30]	2005	Rupture rates of aneurysm surgery	Retrospective cohort	970	Not reported	None	IOR appeared to increase risk of stroke or death (although not statistical focus of paper)
Le Roux [31]	2001	Review of blood transfusion in aneurysm surgery	Retrospective cohort	441	Not reported	None	None
Lin [32]	2013	Factors associated with poor outcome in patients with major intraoperative rupture of cerebral aneurysms	Prospective cohort	647	Not reported	None	Intraoperative rupture is a risk factor for poor outcome
Luostarinen [33]	2015	Frequency of transfusion during aneurysm surgery	Retrospective cohort	488	Not reported	None	Intraoperative RBC transfusion associated with poor neurological outcome
Mahaney [4]	2012	Predictors of postoperative deterioration	Retrospective analysis of RCT	1000	Not reported	None	Logistic regression model - Intentional intraoperative hypotension, blood loss, duration of temporary clip application ≥ 20 min, difficulty of aneurysm exposure were found to associate with poor outcomes
McLaughlin [34]	2004	Analysis of early surgery-related complications	Retrospective single surgeon series	179	80% specialist	None	Surgical complications were associated with worse GOS at 3 months
Molyneux [35]	2002	Trial of clipping versus coiling	RCT	1004	Generalist	None	No relationship between intraoperative complications and outcome
Nguyen [36]	2010	Effect of perioperative hypothermia on occurrence of cardiovascular events in patients undergoing cerebral aneurysm surgery (also from IHAST)	RCT	1000	Not reported	Hypothermia	Hypothermia was not associated with increased occurrence of any single cardiovascular event or composite cardiovascular event
Park [37]	2016	Risk factors for intraoperative rupture of MCA aneurysms	Retrospective cohort	182	Specialist	Mannitol	No statistical inference drawn about outcome
Sandalcioglu [38]	2004	Effect of intraoperative rupture on outcome	Retrospective cohort	169	Not reported	Rupture controlled with double suction, temporary clipping and/or focal tamponade	Rupture has a trend to increase morbidity and mortality when IAR occurs in patients with poor initial condition although this was not statistically significant
Sheth [39]	2014	Effect of intraoperative rupture on vasospasm	Retrospective cohort	500	Not reported	None	Intraoperative rupture was not associated with subsequent vasospasm
Umredkar [40]	2010	Incidence of intracerebral infarcts after aneurysm clipping	Prospective cohort	174	Not reported	None	Longer temporary clipping associated with infarct
Van Lindert [41]	2001	The influence of surgical experience on the rate of intraoperative aneurysm rupture	Retrospective cohort	308	40% by surgeons clipping > 10/year	None	IAR rate higher for non-specialist surgeons
Wester [42]	2009	Single surgeon complications from aneurysm surgery	Retrospective single surgeon series	223	Generalist	None	Intraoperative ruptures decreased with increasing surgical experience
Yamamoto [43]	2017	Effect of perforator infarction after ACOM clipping	Retrospective cohort	104	Not reported	Temporary clipping, Doppler, ICG	Intraoperative rupture and temporary clipping were associated with perforator infarction, perforator infarction associated with poor neurological outcome
Yee [44]	2017	Tranfusion rates in intracranial aneurysm surgery	Retrospective cohort	141	Not reported	None	No comment on outcome
Zhang [45]	2012	Impact of clipping versus coiling in over 60	Retrospective cohort	122	Not reported	None	No statistical relationship between complications and outcome

In 24 of these 36 articles, patients were identified retrospectively from databases or hospital record. In the remaining 12 patients, they were identified prospectively for the purpose of an either RCT or cohort study (Table 1).

### Surgical adverse events

Surgical adverse events were grouped into arterial and non-arterial injuries, incomplete securing of aneurysms, increased operative difficulty and interventions, and these are presented in Table 2.

**Table 2:** Surgical events

Type	Specific complication	Number of papers reporting	Median complication rate	Min	Max
Arterial injury	Arterial injury/occlusion (any)	7	3.8%	1.1%	7.1%
	Arterial injury/occlusion (not further specified)	4	4.6%	1.1%	7.1%
	Arterial occlusion - large vessel	3	2.9%	2.7%	6.1%
	Arterial occlusion - perforator	1	3.8%	3.8%	3.8%
	Arterial stenosis	1	0.6%	0.6%	0.6%
	Distal embolus	1	0.6%	0.6%	0.6%
	Major haemorrhage	1	6.3%	6.3%	6.3%
	Suboptimal clip placement recognised by angiography/Doppler	2	15.5%	11.9%	19.0%
Incomplete securing of aneurysm	Partial occlusion with neck remnant	3	5.4%	3.9%	10.5%
	Partial occlusion with incompletely secure rupture point	2	3.1%	2.8%	3.4%
	Surgery abandoned	1	0.8%	0.8%	0.8%
Increased operative difficulty	Difficult operative exposure	1	35.3%	35.3%	35.3%
	Intraoperative rupture (any stage)	22	16.6%	0.6%	39.1%
	Intraoperative rupture (during exposure)	4	1.1%	0.4%	5.0%
	Intraoperative rupture (during aneurysm dissection)	4	18.6%	2.9%	24.0%
	Intraoperative rupture (during clip application or manipulation)	4	6.7%	0.9%	9.4%
	Parent vessel vasospasm	1	9.1%	9.1%	9.1%
	Swollen brain	3	5.6%	2.8%	8.5%
	Technical failure (non-release of aneurysm clip)	1	0.4%	0.4%	0.4%
Intervention	Administration of local vasodilators	1	1.1%	1.1%	1.1%
	Cerebral angiogram (intraop or within 2 h)	1	9.2%	9.2%	9.2%
	Further neurosurgery (including bypass)	3	0.6%	0.5%	0.6%
	ICP monitor	1	2.9%	2.9%	2.9%
	Lumbar drain	1	31.9%	31.9%	31.9%
	Temporary clipping (any)	9	44.5%	13.2%	76.2%
	Temporary clipping < 10 min	1	27.9%	27.9%	27.9%
	Temporary clipping > 20 min	1	5.8%	5.8%	5.8%
	Temporary clipping 10–20 min	1	10.4%	10.4%	10.4%
	Temporary clipping of ACA	1	21.6%	21.6%	21.6%
	Temporary clipping of ICA	1	8.9%	8.9%	8.9%
Non-arterial injury	Cranial nerve injury	1	2.2%	2.2%	2.2%
	CSF leak	1	1.1%	1.1%	1.1%
	Derangement of neuromonitoring	1	24.1%	24.1%	24.1%
	Direct trauma to brain parenchyma	2	0.6%	0.6%	0.6%
	Eye injury	1	0.6%	0.6%	0.6%
	New subarachnoid haemorrhage or ICH	2	1.0%	0.4%	1.6%

**Fig. 2:**
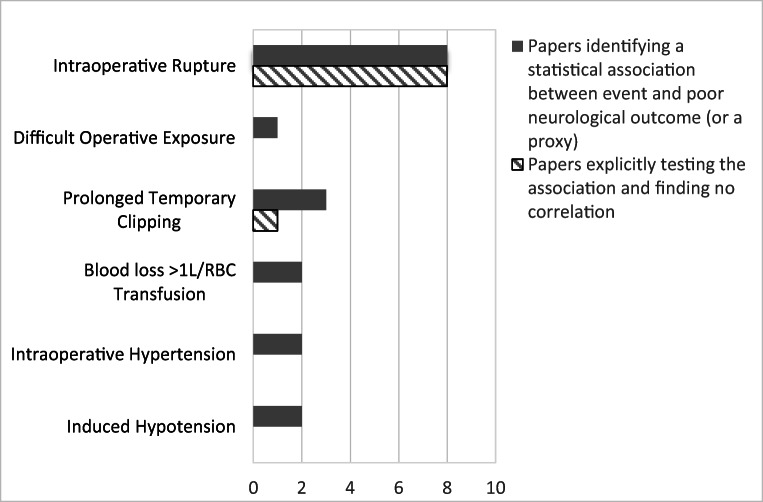
Intraoperative events found to be statistically associated with poor outcome

By far, most commonly reported adverse event was intraoperative rupture which was reported by 22 of the publications, with a median occurrence rate of 16.6% (Table 2). A total of 16 authors reported formally significance testing whether rupture was predictive of poor outcome—these were evenly split with 50% finding an association and the other 50% finding no association between intraoperative rupture and poor outcome (Fig. 2).

The most commonly reported injuries were to the intracranial arteries with rates varying from 1.1 to 7.1% with this variation due at least in part to variability in rates of routine check angiography between centres (Table 2). Causes of increased operative difficulty included surgical exposure (35.4%), swollen brain (5.6%) and intraoperative rupture (16.6%) (Table 2). One author reported an association between challenging operative exposure and poor outcome (Fig. 2).

Intraoperative angiography or Doppler may significantly reduce arterial injury rates as clips were repositioned in as many as 11.9–19% of cases reported their use. Nonetheless, the rate of arterial injury was as high as 7.1% in one series when all patients were exposed to control angiography [16]. Intraoperative rupture is the most well-known complication of surgery for ruptured intracranial aneurysms and accordingly the focus of many of the papers we returned, with 25 reporting on rupture. Definitions of rupture are known to vary widely and this is likely to account for the variation in rupture rates (0.6–39.1%) (Table 2). Several authors presented rupture rates divided by operative stages and we have included those separately. The median rates were 1.1% during exposure, 15.2% during aneurysm dissection and 6.7% during clip application or manipulation (Table 2).

Temporary clipping was the most commonly reported additional surgical intervention and was used as often as 76.2% of the time in one series [17]. Temporary clipping was used both pre-emptively to soften aneurysms for dissection and reactively to control haemorrhage. Length of temporary clipping was found by many authors to be associated with poor outcome and we present separately the duration of clipping by one author reporting a large series where 27.9% of patients underwent temporary clipping for < 10 min, 10.4% for 10–20 min and 5.8% for over 20 min. Three papers found a statistical association between prolonged temporary clipping and poor outcome (Fig. 2), although one tested this and found no association.

### Systemic adverse events

Physiological derangements and interventions that would ordinarily lie outside of the neurosurgical field are presented together in Table 3.

**Table 3:** Systemic events

Type	Specific complication	Number of papers reporting	Median complication rate	Min	Max
Intervention	Non neurosurgical procedure (retrograde jugular venous catheter, reintubation, cardioversion)	2	1.1%	0.6%	1.6%
	Tranfusion of FFP	2	2.0%	0.9%	3.1%
	Tranfusion of platelets	1	1.2%	1.2%	1.2%
	Transfusion of RBC	3	17.7%	7.6%	27.2%
	Unplanned administration of mannitol	1	1.7%	1.7%	1.7%
	Unplanned administration of neuroprotective drugs	1	15.5%	15.5%	15.5%
Physiological derangement	Anaemia	1	3.8%	3.8%	3.8%
	Cardiac event or instability	2	3.9%	0.6%	7.3%
	Electrolyte or glucose disturbance	1	1.3%	1.3%	1.3%
	Hypertension (intended)	1	2.5%	2.5%	2.5%
	Hypertension (unintended)	2	4.1%	2.8%	5.5%
	Hypotension (intended)	3	4.6%	3.3%	44.6%
	Hypotension (unintended)	2	4.3%	3.7%	5.0%

The most commonly reported systemic adverse events were red cell transfusion (17.7%) and unplanned administration of neuroprotective drugs (15.5%) (Table 3) although, notably, one series reported a very high rate of induced hypotension (44.6%) [50]—presumably as a strategy to mitigate the effects of intraoperative rupture.

Intraoperative hypertension, induced hypotension and transfusion were all reported to associate with poor outcome (Fig. 2).

### Neurological adverse events

Neurological harm occurred commonly. Radiologically confirmed infarcts, clinical strokes and undefined neurological deteriorations were all reported and presented in Table 4. In one paper that reported all-cause postoperative neurological deterioration, this occurred in 42.6% of patients [4]. The median rate of radiological stroke was 31.7%, which was significantly higher than the rate of clinical stroke (8.8%) (Table 4).

**Table 4:** Neurological events

Type	Specific complication	Number of papers reporting	Median complication rate	Min	Max
Neurological harm	Clinical stroke (immediate)	2	8.8%	7.9%	9.8%
	Death	1	0.4%	0.4%	0.4%
	Postoperative neurological deterioration (any cause)	1	42.6%	42.6%	42.6%
	Radiological stroke (any)	3	31.7%	4.4%	32.4%
	Radiological cortical stroke	1	15.4%	15.4%	15.4%
	Radiological perforator stroke	1	23.1%	23.1%	23.1%

## Discussion

### Principle findings

Surgery for ruptured intracranial aneurysms is associated with a wide range of adverse events as demonstrated by the range of modalities reported by the authors included in this review.

The adverse surgical conditions encountered by neurovascular surgeons, which include difficult surgical exposure (35.4%), swollen brain (5.6%) and intraoperative rupture (16.6%), all increase the operative difficulty of the procedure (Table 2). It is likely that all of these contribute to the high rate of arterial injury (4.6%), direct parenchymal injury (0.6%) and prolonged temporary ischaemic time (> 10 min in 16.2% of patients) (Table 2). These in turn contribute to the high rate of new postoperative radiological infarction (31.7%) seen in these patients (Table 4). Despite the high rate of intraoperative adverse events, only rupture, difficult exposure and prolonged temporary clipping were found to associate with poor outcome by any of the authors in our series (Fig. 2).

On-table Doppler, indocyanine green (ICG) angiography and digital formal angiography were all used to mitigate vascular injury and confirm aneurysm occlusion. Whilst ICG angiography is now regarded as the gold standard in many units, it was only reported to be used universally by one author [43]. This may be a reflection the fact that many of the series reported in this paper are already relatively historic or simply that the authors of these papers did not feel it was important to explicitly mention in their papers.

Intraoperative rupture was the most commonly reported adverse event but there were discordant results when statistical testing was applied regarding whether it affected neurological outcome (Fig. 2). Whilst a rupture during the final stages of aneurysm dissection when proximal control has been obtained, it is certain that an early rupture with bleeding that is difficult to control or requires prolonged ischaemic time with the application of temporary clips will worsen outcome and this is likely reflected in the associations that were identified between induced hypotension, prolonged temporary clipping and transfusion. Higher rupture rates were associated with less experienced surgeons [41, 42]. Despite it being the most commonly reported adverse event, the only adjunct widely reported to manage intraoperative rupture was the use of temporary clipping in these larger series. Prolonged temporary clipping was associated with poor neurological outcomes, and neuroprotective measures and hypothermia were tried by several authors to mitigate this [4, 5]. Microdialysis has previously demonstrated that a fall of brain tissue PO_2_ to less than 8 mmHg for longer than 30 min in any arterial territory is strongly predictive of cerebral infarction [51]. Further techniques for managing intraoperative rupture that were not described in these series include cardiac standstill with adenosine [52] or rapid ventricular pacing [53]. Whilst it is possible that experienced neurosurgeons are able to mitigate the impact of rupture [13] reducing its impact on patient outcome, pre-dissection ruptures or ruptures that extend into the aneurysm neck are particularly hard to manage and almost certainly associated with a poor prognosis [42, 54]. Many authors advocate the use of endoscopic approaches to better visualize vessel branches and reduce the rate of inadvertent injury [55, 56] but the endoscope offers no advantage over the microscope for control of intraoperative rupture.

### Comparison with other studies

Comparing the intraoperative adverse events in our review with those of the aneurysm series published before, our 2-decade period of capture yields a mixed message of progress. Whilst intraoperative rupture remains commonly reported, its effect on outcome is contentious, and certainly none of the modern studies in our review reported the 3-fold increased rate of unfavourable outcome reported by Batjer and Samson [57]. Conversely, the rates of arterial injury are if anything higher than historical series—Sundt [58] for example reported a major vessel injury rate of 0.62% and perforator injury in 1.4% of cases (versus 2.9% and 3.8% respectively in this review (Table 2)), although it is likely that an increased use of postoperative imaging is leading to the higher rates in our series. The principle surgical strategy for managing rupture (application of temporary clips) has remained popular over this time [57, 59].

It is worth also drawing comparison with the single other large review in the literature covering complications of cerebral aneurysm surgery which bears detailed comparison with this one. Wong et al. [60] review complications reported by in 19 papers covering open cerebrovascular surgery with a total of 7562 patients in total principally undergoing surgery for ruptured or unruptured aneurysms. They identify five of the same intraoperative events as our review—stroke, intraoperative rupture, incomplete aneurysm obliteration, major vessel occlusion, failure to secure rupture site, and the rates of these are similar to those we found—although our review reports a further 45 types of event. This difference is due to the following three factors: (i) we include approximately twice as many patients across the papers we identified, (ii) the focus of this review is more targeted (looking just at intraoperative complications in ruptured aneurysm surgery) resulting in a more granular reporting (e.g. separate rates for perforator and cortical stroke, where this is rolled up into a single complication in the other review) and (iii) we use a wider definition of adverse event meaning that rates of adverse conditions, such as brain swelling as well as interventions such as temporary clipping, are included in our review but excluded from Wong et al.

### Limitations

It would be important to know whether the rate of adverse events in aneurysm surgery has decreased over the 20-year period of our literature review. Modern techniques, such as on-table indocyanine green angiography as well as the increasing concentration of vascular cases in the hands of specialist neurovascular surgeons, may be expected to have had a significant effect on adverse event rates. Unfortunately, the papers we have collected do not lend themselves to a meaningful analysis of this question due to differences in reporting standards, frequently many years of data capture within each paper and confounders, e.g. single author series where we might expect a reduction in event rates over time with greater operator experience [5, 34].

Analysis of the clinically relevant complications that occur during clipping is made particularly challenging by the heterogenous patient group: the ultimate impact of cerebral insult on patient outcome is difficult to correlate when the outcome itself is difficult to predict. To further confound this, the overwhelming majority of events were only reported by a few authors. With the exception of rupture (22 authors), temporary clipping (9 authors) and arterial injury (7 authors), the other event rates (e.g. brain swelling, parent vessel vasospasm or cranial nerve injury) were all reported by fewer than 5 authors.

Similarly due to the heterogeneity of the date reported in these papers, the data did not lend themselves to analysis of particular patient subgroups which may have been significant, e.g. we might have expected higher rates of brain swelling rates in patients with associated haematomas with mass effect and lower rates of retraction injury in patients with middle cerebral artery aneurysms due to their relative accessibility.

There are a wide range of adverse event rates reported in the literature and this often reflects differences in definition (e.g. many authors reported rupture as an adverse event only if it occurred in a pre-dissection phase or before proximal control could be obtained, whereas others reported rupture at any stage). For the interventions, in particular, surgeon preference played a large part—with some surgeons choosing to use temporary clips almost routinely as a softening strategy even for uncomplicated aneurysms—whilst others typically deployed them only as an emergency measure to gain vascular control. These differences place a natural limit on the utility of reporting adverse events simply as the raw rate.

As our search strategy returned papers that focused on “adverse events” or “complications”, this study, interventions which may not have been regarded as meeting the definition of an adverse event by the study author are likely to be underemphasised. Notably, frequency of intraoperative external ventricular drain placement (which presumably occurred commonly) was not reported in any of our series—we hypothesise that this is because most authors would not regard it as an adverse event—although it would have met the wider definition we use in this paper. Similarly, cerebrospinal fluid (CSF) diversion by lumbar drain insertion rates was only commented on in one paper at 31.9% (Table 2). As with temporary clipping, an episode of intraoperative CSF diversion can be indicative of an adverse event resulting in a deviation of surgery from its operative course, or simply a surgeon’s routine practice for the particular case in hand. Being unable to distinguish between these situations was a significant limitation of the approach we have taken in this paper.

Moreover, whilst challenging aneurysm morphology and severe brain swelling are widely believed to significantly increase the risks associated with aneurysm surgery, these were only rarely reported by authors in our review. It seems likely that events that exist on a spectrum (such as degree of brain swelling) and which are consequently more difficult to report may have been relatively under-reported.

## Summary

Whilst endovascular techniques have become increasingly used in open aneurysm surgery, open surgical repair remains the preferred modality for many aneurysms [2, 3]. Compared with the fast pace of innovation seen in endovascular techniques for coiling, only a small number of new technologies have been adopted in open aneurysm surgery since Yasargil developed the microscopic approaches used by the majority of surgeons today [61]. There is considerable morbidity that is associated with open repair of cerebral aneurysms attributable to technical surgical challenges. The overwhelmingly most commonly reported surgical adverse event is intraoperative rupture and it is certain that an uncontrolled rupture (occurring either during pre-dissection or from a tear in the neck of the aneurysm) leads to poor outcomes for many patients. Despite this, the papers in this review were split when trying to show whether there was a statistical association between rupture and poor outcome. This underscores the point that beyond surgeons’ experience and intuitions, we do not have an evidence-based understanding of the drivers of morbidity in aneurysm surgery. No papers, for example, demonstrated an association between brain swelling and poor patient outcome, despite the surgical intuition that this increase in the difficulty of the surgical conditions is highly likely to impact upon the morbidity of the surgery. Further research is needed into this topic and would help by a consensus on the definitions of each intraoperative events to enable comparison between patient series. A better understanding of the relationship between the adverse events of aneurysm clipping and their contribution to patient morbidity would not only help target further research to address these problems but also help surgeons mitigate the impact of those events when they occur.

## Electronic supplementary material

ESM 1 (XLSX 137 kb).
